# Metal protoporphyrin‐induced self‐assembly nanoprobe enabling precise tracking and antioxidant protection of stem cells for ischemic stroke therapy

**DOI:** 10.1002/SMMD.20220037

**Published:** 2023-02-14

**Authors:** Yimeng Shu, Hui Shen, Minghua Yao, Jie Shen, Guo‐Yuan Yang, Hangrong Chen, Yaohui Tang, Ming Ma

**Affiliations:** ^1^ State Key Laboratory of High Performance Ceramics and Superfine Microstructures Shanghai Institute of Ceramics Chinese Academy of Sciences Shanghai China; ^2^ Center of Materials Science and Optoelectronics Engineering University of Chinese Academy of Sciences Beijing China; ^3^ Med‐X Research Institute and School of Biomedical Engineering Shanghai Jiao Tong University Shanghai China; ^4^ Department of Ultrasound Shanghai General Hospital School of Medicine Shanghai Jiao Tong University Shanghai China; ^5^ School of Chemistry and Materials Science Hangzhou Institute for Advanced Study University of Chinese Academy of Sciences Hangzhou China; ^6^ Ankerui (Shanxi) Biological Cell Co., Ltd. Xiaohe Industrial Park Comprehensive Reform Demonstration Zone Taiyuan China

**Keywords:** cell labeling, ischemic stroke, self‐assembly, stem cell therapy, theranostic

## Abstract

Mesenchymal stem cell (MSC)‐based therapy has provided a promising strategy for the treatment of ischemic stroke, which is still restricted by the lack of long‐term cell tracking strategy as well as the poor survival rate of stem cells in ischemic region. Herein, a dual‐functional nanoprobe, cobalt protoporphyrin‐induced nano‐self‐assembly (CPSP), has been developed through a cobalt protoporphyrin IX (CoPP) aggregation‐induced self‐assembly strategy, which combines CoPP and superparamagnetic iron oxide (SPION) via a simple solvent evaporation‐driven method. Without any additional carrier materials, the obtained CPSP is featured with good biocompatibility and high proportions of active ingredients. The SPIONs in CPSPs form a cluster‐like structure, endowing this nano‐self‐assembly with excellent T_2_‐weighted magnetic resonance (MR) imaging performance. Furthermore, the CoPP released from CPSPs could effectively protect MSCs by upregulating heme oxygenase 1 (HO‐1) expression. The in vivo cell tracing capacity of CPSPs is confirmed by monitoring the migration of labeled MSCs with MR imaging in a middle cerebral artery occlusion mouse model. More importantly, the sustained release of CoPP from CPSPs improves the survival of transplanted MSCs and promotes neural repair and neurobehavioral recovery of ischemic mice. Overall, this work presents a novel dual‐functional nanoagent with an ingenious design for advancing MSC‐based therapy.

## Graphical abstract

A novel cobalt protoporphyrin‐induced nano‐self‐assembly (CPSP) with magnetic resonance (MR) imaging and antioxidative protection dual‐functions is ingeniously constructed for advancing stem cell therapy. CPSP labeled stem cells can not only be tracked in vivo by MR imaging, but also survive longer in the ischemic lesions, thus achieving improved therapeutic effect in ischemic stroke.

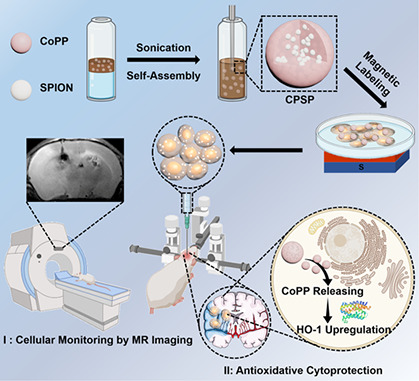

1

**Key Points**

- Cobalt protoporphyrin IX induces the formation of multifunctional labeling nanoagent by self‐assembly without other carrier materials.
- Superparamagnetic iron oxide nanoparticles‐based MR imaging enables stem cell tracking in vivo.
- Antioxidative protection function improves the therapeutic effect of stem cells in ischemic stroke treatment.

## 2 INTRODUCTION

Stroke has been posed as the second largest cause of mortality while the first leading cause of long‐term disability worldwide.^1^ Ischemic stroke accounts for more than 80% of brain hemorrhage. So far, the approved regime includes the administration of tissue plasminogen activator and simultaneous endovascular therapy, which however suffers from their poor efficacy due to the narrow therapeutic time window and risk of hemorrhage.^2^, ^3^ Recently, transplantation of mesenchymal stem cells (MSCs) into ischemic brain has emerged as a promising method to broaden the therapeutic window of stroke and improve both nerve tissue regeneration and functional recovery via a multitude of plausible mechanisms, including cell differentiation, paracrine function, mitochondria transfer, and immunomodulation.^4^, ^5^, ^6^ However, the clinical stem cell therapy is limited by the lack of strategies for precisely tracking engrafted stem cells in vivo and ensuring their survival in ischemic regions.^7^ To understand the fate of MSCs after in vivo transplantation and their biosafety as well as the capacity to promote stroke rehabilitation, an accurate and consecutive in vivo MSC tracking is necessary.^8^ Notably, most of the engrafted MSCs cannot survive post‐transplantation, presumably due to cell apoptosis and anoikis emanating from the harsh ischemic microenvironment, which limit their therapeutic effects.^9^, ^10^ Therefore, the development of MSC imaging nanoprobe with cytoprotective function will be of great significance for the precise tracking of the transplanted MSCs for stroke management.

Magnetic resonance (MR) imaging, as an advanced medical imaging diagnostic technique, has received extensive attention in the field of stem cell therapy.^11^ Owing to its sufficient tissue penetrability, MR can realize imaging of soft tissue as well as provide an insight into the functional information related to tissue micro‐framework non‐invasive yet at a relatively high spatial resolution.^12^, ^13^ When used to monitor the fate of the transplanted stem cells, MR imaging could not only afford the localization of the transplanted stem cells, but also afford anatomical and pathological information of the tissues surrounding stem cells, including edema or inflammation at the transplantation site.^14^ Among the existing MR contrast agents, superparamagnetic iron oxide (SPION) has been extensively employed for stem cell tracking in some clinical trials of transplanted stem cells into humans due to its stable imaging performance as well as its good biocompatibility.^13^ Nevertheless, monodispersed SPIONs exhibit relatively low T_2_‐weighted relaxivity, which may compromise MR imaging sensitivity.^15^ Though several self‐assembly‐based strategies have been explored to aggregate the SPIONs into clusters to improve the MR imaging performance,^13^, ^16^, ^17^, ^18^ they are still hampered by several shortcomings, such as complex multi‐step synthesis, low‐effective ingredient content, undesirable immunogenic response or toxicity due to high carrier‐specific gravity. Additionally, direct engineering of MSCs with cytoprotective agents, including amifostine, generally encounters the problems of short‐acting and inefficient performance.^19^ Similarly, scaffold‐mediated transplantation of stem cells often has difficulties in tuning of biodegradation, which may also cause tissue trauma.^20^ Hence, constructing a multifunctional nanoprobe for stem cell tracking and transplantation can avoid mechanical damage to tissues while ensuring efficiency of cell phagocytosis,^11^ which is of considerable significance for MSC‐based therapy of ischemic stroke management.

**Scheme 1:**
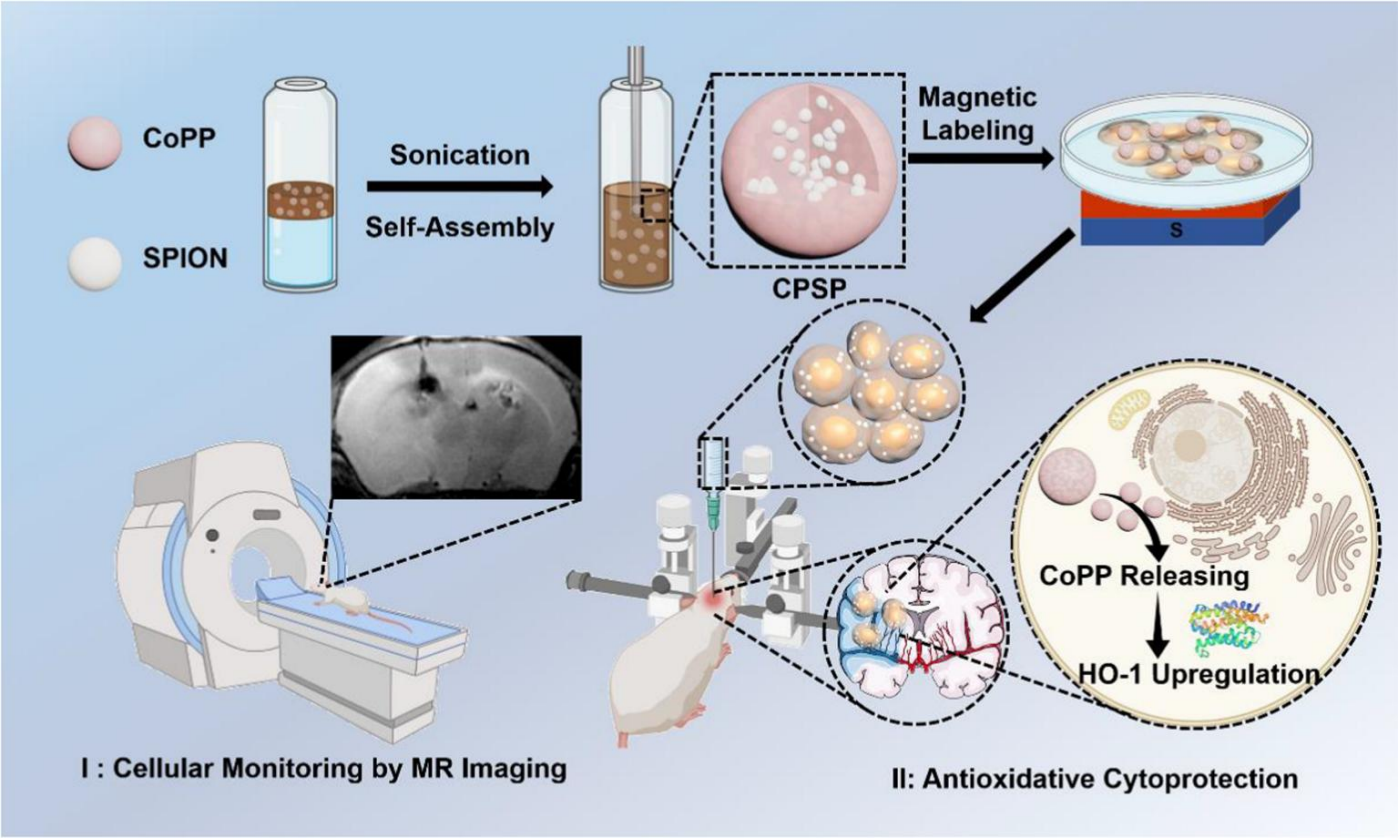
Schematic illustration of the synthesis process of CPSP and its dual functions in MSC‐based ischemic stroke therapy. CPSP, cobalt protoporphyrin‐induced nano‐self‐assembly; MSC, mesenchymal stem cell.

Cobalt protoporphyrin (CoPP), an amphiphilic porphyrin derivative, is well known for its ability of inducing heme oxygenase 1 (HO‐1) and thus can be exploited to protect MSCs against ischemic injury.^21^ Nevertheless, the low solubility of CoPP in the aqueous solution and potential toxicity induced by the excessive CoPP may limit its application for protecting MSCs.^22^, ^23^ In this study, a simple yet efficient CoPP aggregation‐induced self‐assembly strategy was developed to integrate many small‐sized SPIONs into CoPP‐induced self‐assembled nanoparticles (named as CPSP), which could simultaneously enhance the MR imaging performance of SPIONs and protect MSCs against oxidative stress (Scheme 1). Clustering of monodispersed SPIONs in the CPSPs considerably increases the MR relaxivity, which can significantly enhance MR imaging performance for stem cell tracking. More importantly, this oil‐in‐water‐based self‐assembly strategy can convert two poorly water‐soluble components into hydrophilic nanoparticles while avoiding additional carrier materials or surfactants, thereby ensuring high drug loading efficiency and biocompatibility. The CoPP can be slowly released from CPSP and protect the labeled MSCs against oxidative stress. Consequently, these CPSPs could be acted as theranostic nanoagents for MSCs labeling and transplantation for in vivo applications. The CPSP‐labeled MSCs transplanted to the ischemic regions of mice were tracked in vivo by MR imaging, while their capacity to improve the nerve regeneration and neurobehavioral recovery was also evaluated in a mouse middle cerebral artery occlusion model to verify the potential of this nano‐self‐assembly for MSC therapy.

## 3 RESULTS AND DISCUSSION

### 3.1 Preparation and characterization of CPSPs

The monodispersed SPIONs were prepared through a thermal deposition method by using oleic acid as surface ligand (diameter = 5.0 ± 1.0 nm; Figure S1).^24^ The as‐synthesized SPIONs were then simply dissolved along with CoPP at an appropriate proportion in toluene and sonicated in water until the formation of a uniform oil‐in‐water emulsion. Thereafter, a self‐assembled CPSP nanoprobe was obtained by removing the volatile solvent and any unreacted CoPP via vapourization and dialysis, respectively. Notably, the nanoprobe possesses good water solubility even in the absence of an additional surfactant, presumably because the amphiphilic CoPP molecules anchored to the surface of the oleic‐acid‐modified SPION clusters via hydrophobic interactions and thus exposed the hydrophilic carboxyl group toward water, endowing the nanoprobe with good hydrophilicity.^25^

**FIGURE 1:**
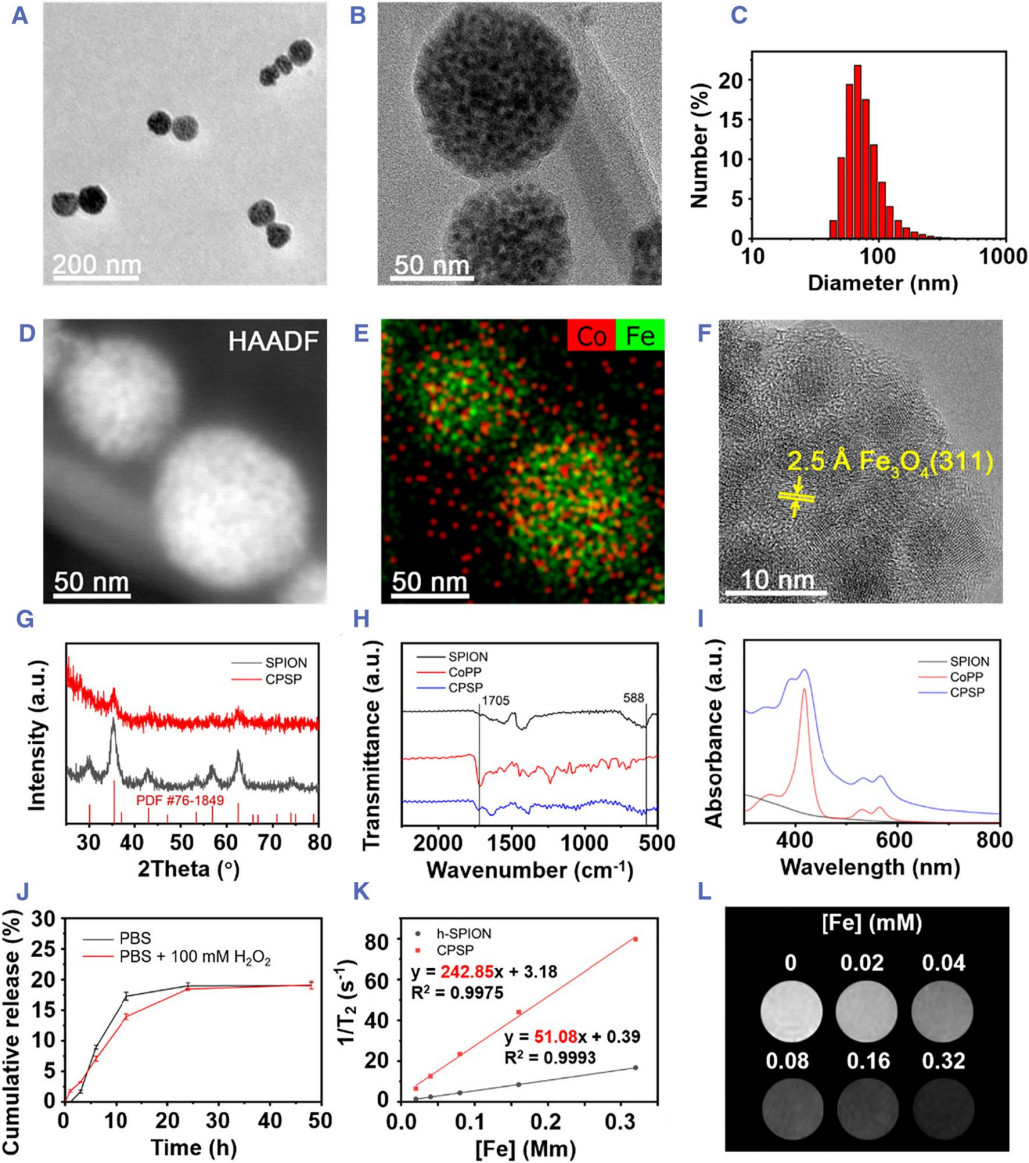
Characterizations of CPSP. (A,B) TEM images of CPSP at different magnifications. (C) Hydrodynamic size distribution of CPSP in water. (D) HAADF image and (E) element mapping analysis of CPSP. (F) HRTEM image of CPSP. (G) XRD patterns of SPION and CPSP. (H) FTIR spectra of SPION, CoPP, and CPSP. (I) UV‐vis spectra of SPION, CoPP, and CPSP. (J) Cumulative release curve of CoPP from CPSPs in PBS in the presence or absence of 100 mM H_2_O_2_. (K) T_2_ relaxation rate of h‐SPION and CPSP. (L) MR images of CPSP aqueous solutions at different concentrations. CoPP, cobalt protoporphyrin; CPSP, cobalt protoporphyrin‐induced nano‐self‐assembly; HAADF, high‐angle annular dark‐field; MR, magnetic resonance; PBS, phosphate buffered saline; SPION, superparamagnetic iron oxide.

As shown in transmission electron microscopy (TEM) and scanning electron microscopy (SEM) micrographs, SPIONs were homogenously wrapped by CoPP and self‐assembled into a cluster‐like structure with a diameter of around 70 nm (Figure 1A,B and Figure S2). Correspondingly, the average dynamic light scattering (DLS) diameters of CPSPs were about 101, 92, 75, and 88 nm in water, phosphate buffered saline (PBS), culture medium, and stimulated body fluid (SBF), respectively, which could have structural stability for up to 7 days (Figure 1C and Figure S3). Furthermore, the zeta potential of the CPSPs was around −21.9 mV owing to the presence of carboxyl groups (‒COOH) in CoPP. The high‐angle annular dark‐field (HAADF) TEM images and elemental mapping analysis showed a strong signal of discretely distributed Fe and Co in the nanoprobe, demonstrating the random distribution of SPIONs and CoPP in the nano‐self‐assembly (Figure 1D,E). The lattice fringes corresponding to (311) crystal planes of Fe_3_O_4_ could be detected in the high‐resolution TEM image (Figure 1F). Moreover, the main peaks of SPIONs at 35° and 62° corresponding to the (311) and (440) crystal planes of SPIONs could be identified in the XRD pattern of CPSPs, indicating that the SPIONs could maintain a good crystal structure during the self‐assembly process (Figure 1G). The FTIR spectra of CPSP display the bands at 1705 and 588 cm^−1^ are corresponding to the carbonyl (C=O) groups in CoPP and Fe_3_O_4_, respectively, further demonstrating the co‐existence of CoPP and SPIONs in CPSPs (Figure 1H). Moreover, the valence state of cobalt in CPSPs was analyzed by the X‐ray photoelectron spectroscopy (XPS), where the peaks at 780.6 and 796.0 eV are assigned to the 2p3/2 and 2p1/2 of Co(II) (Figure S4).

To gain further insight into the self‐assembly mechanism of SPIONs and CoPP, the ultraviolet‐visible (UV‐vis) spectra of CPSPs under various conditions were evaluated. Besides the characteristic peaks of CoPP, the peak at 392 nm in the UV‐vis spectra of CPSPs is resulted from the blue‐shift of Soret band absorption of CoPP (417 nm), which could be ascribed to the H‐aggregation of CoPP during the self‐assembling via π‐π stacking interaction (Figure 1I).^26^ As shown in Figure S5, the UV‐vis spectra of CPSPs in sodium dodecyl sulfate (SDS, 0.2%) or DMSO exerted significant changes as compared with that of in water, indicating the existence of hydrophobic interaction between CoPP and SPIONs.^27^ Interestingly, the turbidity of the CPSP decreased slightly with an increase in the concentration of NaCl (implying increased ionic strength) probably due to the disassembly of CPSP, suggestive of the electrostatic interactions between CoPP and SPIONs (Figure S6).^28^ Altogether, the self‐assembly of CPSP is considered to be mainly attributed to an integrated mechanism of hydrophobic, π‐π stacking, and electrostatic interactions between CoPP and SPIONs.

The exact proportions of CoPP and SPIONs in CPSP can be calculated from the inductively coupled plasma (ICP) data, which are found to be 32.4% and 67.6%, respectively. Moreover, the release behavior of the CoPP from CPSPs shown in Figure 1J indicates that the cumulative released amount of CoPP could both reach up to 19% in the presence or absence of H_2_O_2_ in the first 24 h, and no sudden release would occur.

### 3.2 MRI performance of CPSPs

Aggregating magnetic particles into clusters is considered as a fruitful method to enhance their T_2_‐weighted MR imaging performance, which can significantly shorten the T_2_ relaxation time thereby increasing the transverse molar relaxivity (*r*_2_).^29^ The clusters composed of magnetic particles can generate highly inhomogeneous magnetic field and lead to the loss of phase coherence once proton diffuse around the clusters, thereby shortening the relaxation time. Considering that a large number of superparamagnetic particles were wrapped in clusters, CPSPs were expected to have excellent MR imaging performance. In order to evaluate the improvement of MR imaging performance by self‐assembling strategy, a reference hydrophilic SPION (named as h‐SPION) with a hydrated particle size of around 22 nm was synthesized for comparison (Figures S7 and S8).^30^ The relaxation time of CPSPs and h‐SPIONs at various Fe concentrations was acquired by a 0.5 T MR scanner. As shown in Figure 1K, CPSPs displayed the *r*_2_ value of 243 mM^−1^·s^−1^, significantly higher than that of the reference h‐SPIONs (51 mM^−1^·s^−1^) and also much higher than several clinically approved Fe‐based MR contrast agents (e.g., 72 mM^−1^·s^−1^ for Ferumoxsil, 98.3 mM^−1^·s^−1^ for Ferumoxide, 150 mM^−1^·s^−1^ for Resovist, etc.). Moreover, CPSPs showed a dose‐dependent darkening effect in the concentration range of 0.02–0.32 mM, demonstrating the potential of CPSPs to trace MSCs in vivo even at low doses (Figure 1L).

### 3.3 MSC labeling and cytotoxicity of CPSPs

**FIGURE 2:**
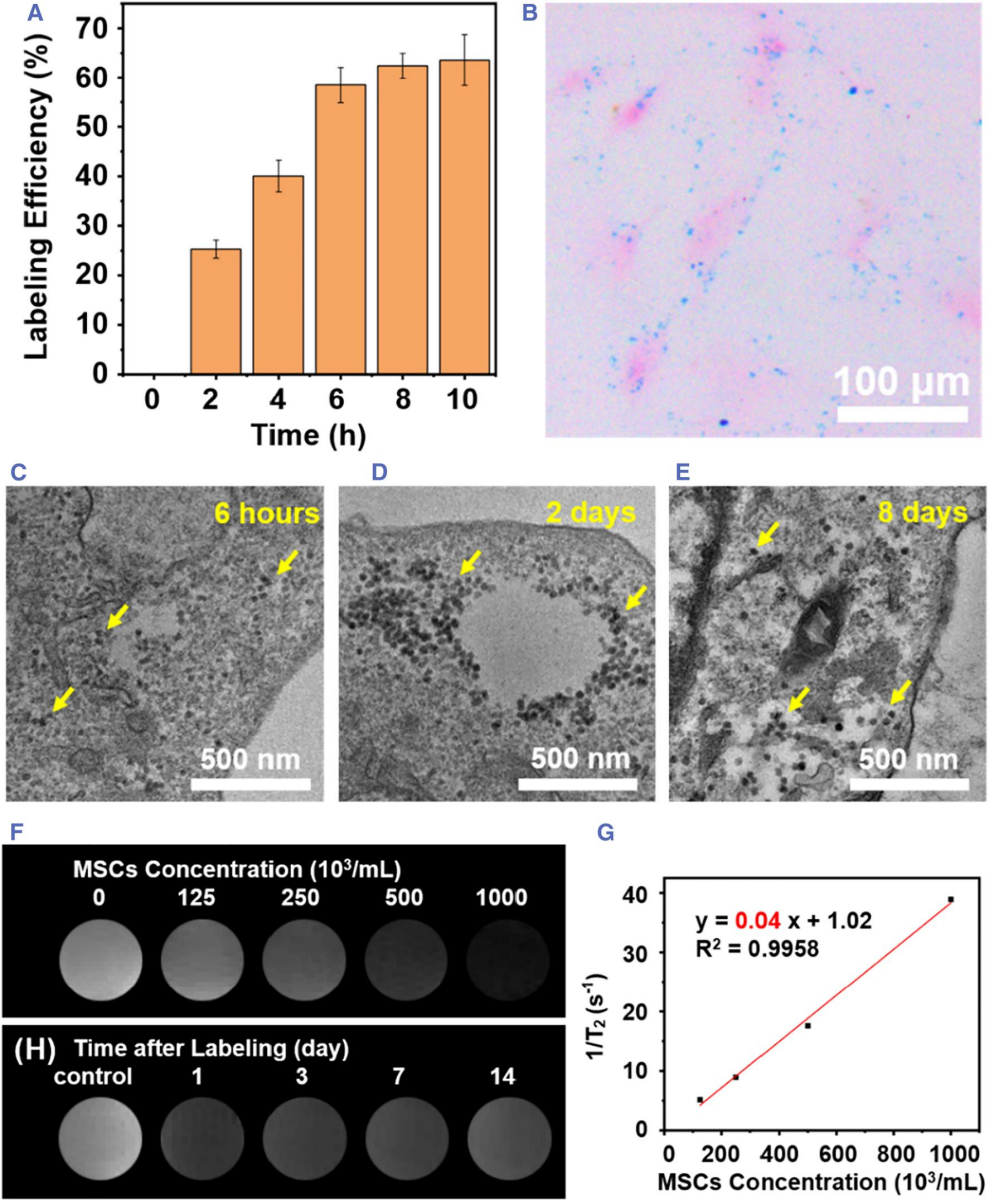
In vitro labeling of MSCs with CPSP. (A) Labeling efficiency of CPSP with different co‐incubation time evaluated by ICP‐OES. (B) Representative Prussian blue staining image of MSCs labeled with CPSP. (C–E) TEM images of MSCs (C) 6 h, (D) 2 days, and (E) 8 days after labeled with CPSP. The yellow arrows indicate CPSPs internalized by MSCs. (F,G) In vitro MR images of the PBS solution containing MSCs labeled with 5 μg/ml CPSPs at different concentrations (F) and the corresponding T_2_ relaxation rate (G). (H) In vitro MR images of the PBS solution containing MSCs at the concentration of 5 × 10^5^ cells/mL at different times after being labeled with 5 μg/ml CPSPs. CPSP, cobalt protoporphyrin‐induced nano‐self‐assembly; ICP‐OES, inductively coupled plasma optical spectrometry; MR, magnetic resonance; MSC, mesenchymal stem cell; PBS, phosphate buffered saline; TEM, transmission electron microscopy.

To ensure that the MRI signal of CPSPs can accurately reflect the position of transplanted MSCs in the cortical tissues, the feasibility of labeling MSCs with CPSPs was confirmed by the analysis of ICP‐OES, Prussian blue staining, and bio‐electron microscopy images. The ICP‐OES results indicated that the proportion of MSCs internalizing CPSPs was gradually increased to 62% within 6 h of incubation with 30 μg CPSPs per well (5 μg/ml CPSP for about a total 1.0 × 10^6^ cells) and remained no obvious changes with further increase of incubation time (Figure 2A). Thus, a time period of 6 h was chosen as an optimum labeling time, while the corresponding average amount of CoPP in each MSC was calculated to be 1.74 × 10^−14^ mol, which was proven to be sufficient to exert antioxidant effect.^31^ Under the optimized culture condition, the Prussian blue staining image clearly shows the presence of blue particles in each of the CPSPs‐treated cell (Figure 2B), indicative of efficient cellular internalization of the CPSPs. Furthermore, as shown in TEM images (Figure 2C–E), a considerable amount of CPSPs displaying a spherical structure were observed inside the MSCs within 8 days, implying strong resistance to lysosomal degradation and the long‐term MSC tracing function of CPSPs. To evaluate the potential of CPSP‐labeled MSCs for MR tracking in vitro, the MR images of labeled MSCs were acquired at different cell concentrations and labeling time. As shown in Figure 2F,G, a concentration‐dependent darkening effect can be clearly observed, and the reciprocal of relaxation time 1/T_2_ has good regressive relation with the concentration of MSCs (*R* ^2^ = 0.9958), enabling to estimate the number of cells from the MR imaging signal. The MR imaging signal almost remained unchanged for 14 days after incubation, implying that CPSPs have the capability of long‐term stable trackers of MSCs (Figure 2H).

**FIGURE 3:**
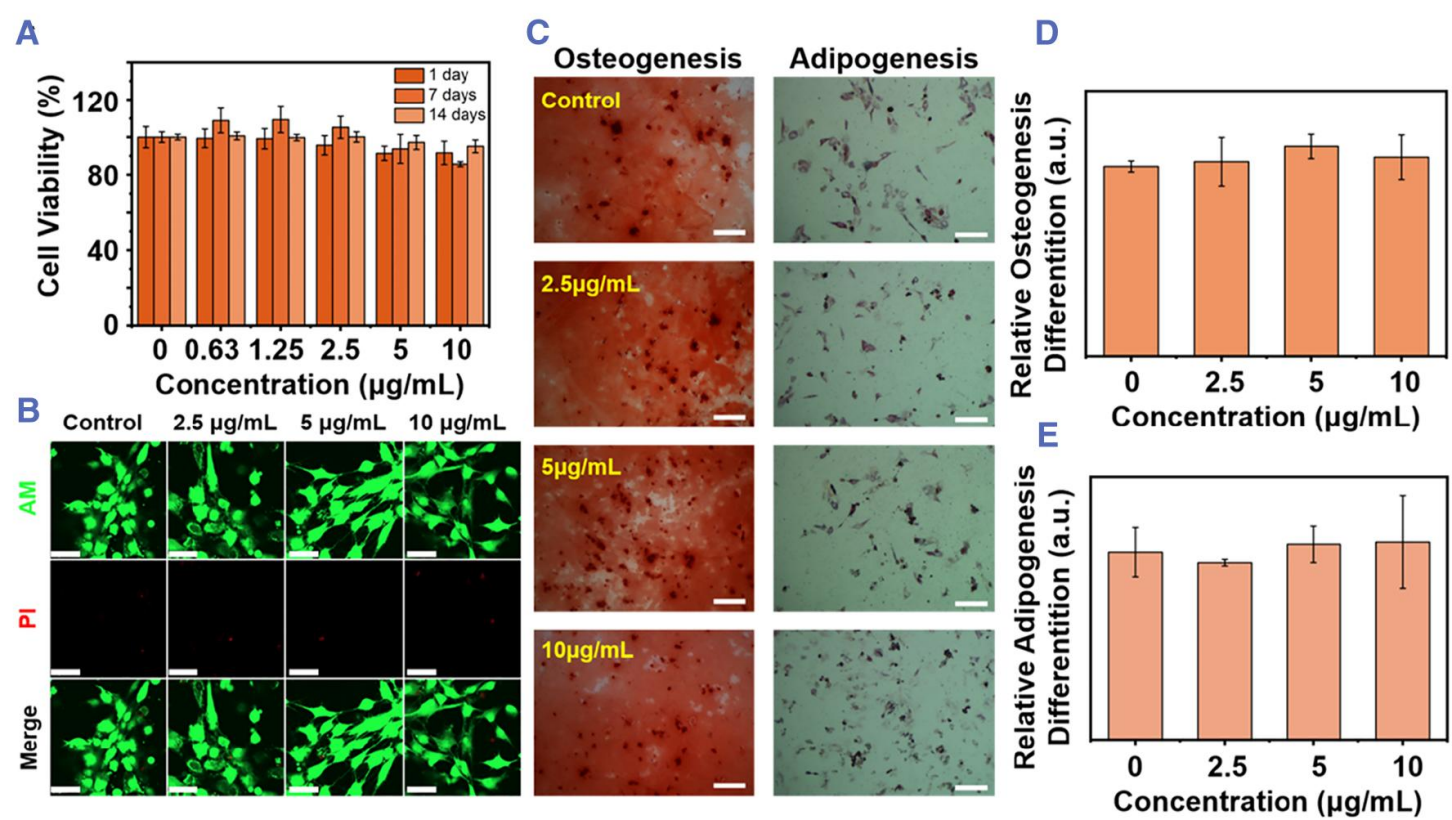
Effects of CPSP labeling on the viability and pluripotency of MSCs. (A) Relative cell viability of MSCs labeled with different concentrations of CPSPs at days 1, 7, and 14 (*n* = 5). (B) Fluorescence microscopy images of calcein AM/PI‐stained MSCs after co‐incubation with CPSPs at different concentrations at day 1. Scale bar, 100 μm. (C) Osteogenesis and adipogenesis of unlabeled MSCs and MSCs labeled with CPSPs at different concentrations. Scale bar, 200 μm. (D) Absorbance values of the dissolved calcium deposits in MSCs at 562 nm at day 14 of osteogenesis induction. (E) Absorbance values of the dissolved lipid droplets in MSCs at 490 nm at day 14 after adipogenesis induction. CPSP, cobalt protoporphyrin‐induced nano‐self‐assembly; MSC, mesenchymal stem cell.

Furthermore, the cytotoxic effect of CPSPs on MSCs for 1, 7, and 14 days was evaluated separately by cell counting kit 8 (cck‐8) assay at different concentrations. As shown in Figure 3A, the viability of cells treated with different concentrations of CPSPs shows no significant differences from the unlabeled MSCs. Similarly, the MSCs labeled with the different concentrations of CPSPs were stained by the calcein AM/propidium iodide (PI) double stain kit at day 1. As can be seen from Figure 3B, similarly to the control group, all the experimental groups show very little red fluorescence, indicative of only a few numbers of apoptotic cells. Altogether, the above results indicate the good viability of labeled MSCs even for up to 10 μg/ml of CPSPs content.

Since MSCs can differentiate into several types of cells, such as osteoblasts and adipocytes, we next evaluated if the labeling could affect their pluripotency, which is an important evaluation criterion for the biosafety of materials in stem cell therapy. As shown in Figure 3C, both the unlabeled MSCs and MSCs labeled with CPSPs at different concentrations could generate red calcified and red lipid droplets after 14 days of osteogenesis and adipogenesis induction as evaluated by azarin red staining and oil red O staining, respectively. The calcium nodules were further quantified by using a UV‐vis spectrophotometer once dissolved by cetylpyridine chloride (CPC) and showed no significant difference between all groups, indicating similar osteogenic differentiation ability of labeled or unlabeled MSCs (Figure 3D). Similarly, the lipid droplets in all groups were dissolved in isopropanol and quantified according to their absorbance values at 490 nm, which had no significantly differences either, indicating the negligible influence of CPSPs on the adipogenic differentiation of MSCs (Figure 3E). In summary, CPSPs at concentration lower than 10 μg/ml had almost no adverse effects on the viability and differentiation ability of BMSCs, indicative of their safety in stem cell therapy.

### 3.4 Cytoprotection capacity of CPSPs

**FIGURE 4:**
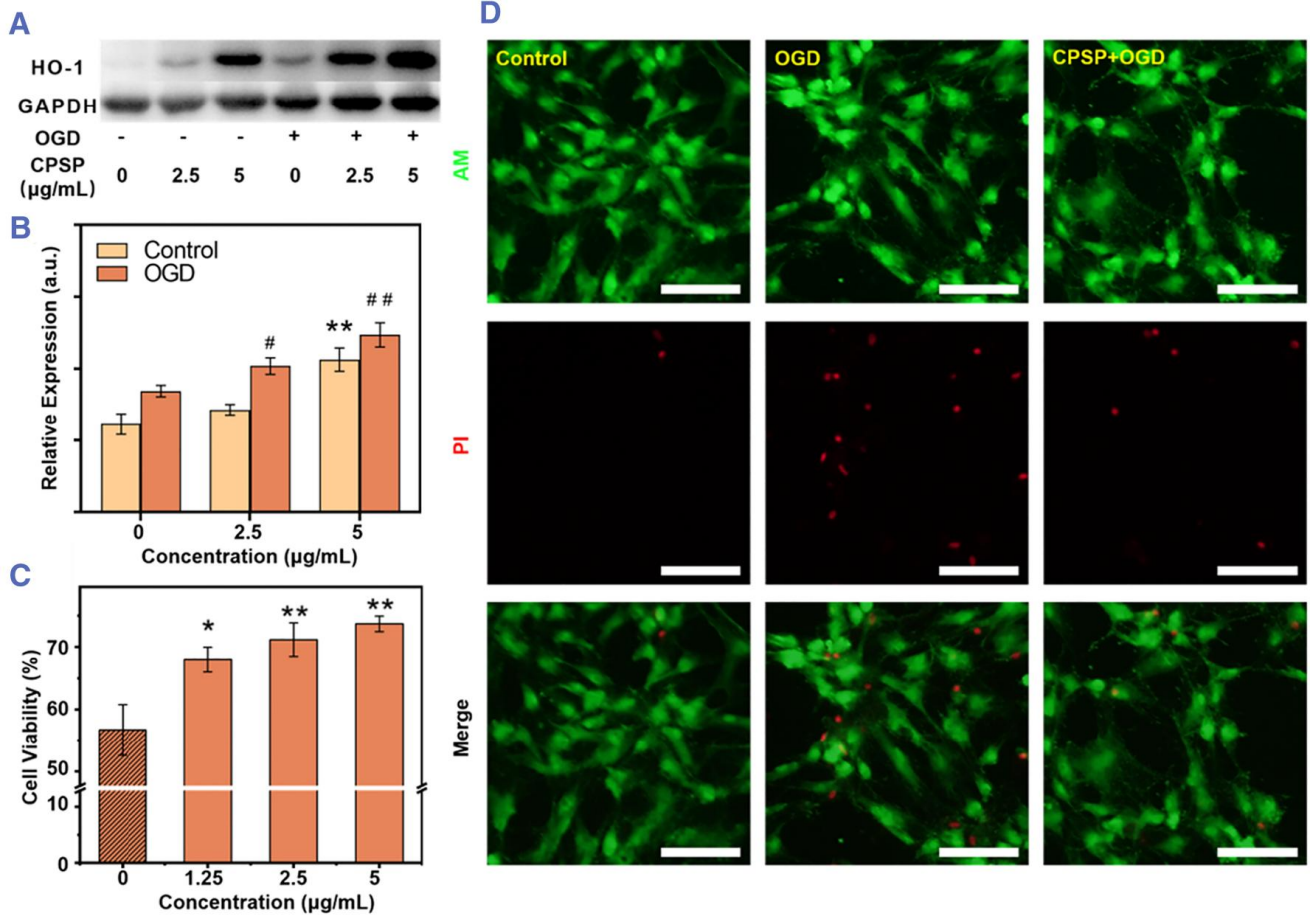
CPSP improved MSCs survival in vitro. (A) Western blot images and (B) the corresponding HO‐1 expression level of MSCs without any treatment or with different treatments (labeling with 2.5 or 5 μg/ml CPSPs alone, OGD alone, or OGD after labeling with 2.5 or 5 μg/ml CPSPs) (*n* = 4). **p < 0.01 versus no treatment group; ^#^ *p* < 0.05; ^##^ *p* < 0.1 versus OGD alone group. (C) Cell viability of CPSP‐labeled MSCs after OGD treatment as evaluated by using CCK‐8 assay (*n* = 4). (D) Cell apoptosis of CPSP‐labeled MSCs after OGD treatment as evaluated by annexin/PI staining. Scale bar, 100 μm. CPSP, cobalt protoporphyrin‐induced nano‐self‐assembly; HO‐1, heme oxygenase 1; MSC, mesenchymal stem cell; OGD, oxygen and glucose deprivation.

The poor survival of transplanted MSCs in the ischemic area generally poses a huge obstacle for the stem cell therapy. Previous studies have demonstrated that over 80% of transplanted cells die within 72 h after injection, presumably because of cell death or apoptosis triggered by the ischemic microenvironment.^9^ Fortunately, CoPP, as an important metalloporphyrin, could be used to protect stem cells against ischemia injury through activation of the ERK/COX‐2 or ERK/NRF2 signaling pathway, and enhancement of the HO‐1 expression.^32^ Although the stem cell protective function and mechanism of CoPP were investigated in previous reports, whether CoPP‐induced nano‐self‐assembly had a similar cytoprotective effect yet remaining unexplored. Thus, the expression levels of HO‐1 of MSCs labeled with different concentrations of CPSP (0, 2.5, and 5 μg/ml) by western blotting analysis were conducted, where an oxygen and glucose deprivation (OGD) model was employed to mimic ischemic lesion microenvironment in vivo. As shown in Figure 4A, the HO‐1 expression level of MSCs obviously increase after OGD treatment, which could be attributed to the response of MSCs against an excessive ROS produced by OGD. More importantly, the HO‐1 expression of MSCs showed a significant and dose‐dependent increase after labeling by CPSPs. Quantitatively, the HO‐1 protein expression of MSCs was upregulated by 1.21 and 1.46 folds at CPSP concentrations of 2.5 and 5 μg/ml, respectively, than that of their unlabeled counterparts (Figure 4B). Furthermore, as shown in Figure S9, the HO‐1 expression level of MSCs shows a gradual increase after treatment with CPSPs for up to different time points, suggesting a significant effect of CPSP on HO‐1 induction of MSCs.

Afterward, the improvement of CPSPs labeling on MSC survival in ischemic environment was evaluated by the OGD model as well as hydrogen peroxide (H_2_O_2_)‐induced oxidative stress model in vitro. The viability of the MSCs was reduced to around 56.7% after OGD stimulation, and the CPSPs were found to induce a dose‐dependent improvement on the viability of the labeled MSCs. In detail, after being labeled with 1.25 and 2.5 μg/ml CPSPs, 68.0% and 71.2% MSCs could maintain viability, respectively, while 73.7% MSCs were found to be viable when labeled with 5 μg/ml CPSPs (Figure 4C). Similarly, after exposure to 200 μM H_2_O_2_ for 6 h, the viability of unlabeled MSCs was found to be 55%, which rose to 68% in cells labeled with the 5 μg/ml of CPSPs (Figure S10). Furthermore, the apoptosis of MSCs in OGD model was obviously reduced in the CPSP‐labeling group as revealed by using annexin/PI staining (Figure 4D). These results showed that death of cells induced by the oxygen and glucose deprivation could be efficiently prevented from apoptosis in vitro via their labeling by CPSPs and consequent release of CoPP. To further verify whether the CPSPs could also protect transplanted MSCs in vivo, the unlabeled and CPSP‐labeled MSCs were stained with Dil and injected into the ischemic mice brains, respectively. The TUNEL/DAPI staining of brain sections clearly showed that the apoptotic rate of transplanted MSCs significantly decreased with CPSP labeling as compared to the unlabeled MSCs, indicating that CPSP labeling also improved the survival of MSCs after transplantation (Figure S11).

### 3.5 In vivo MR monitoring of CPSP‐labeled MSCs

**FIGURE 5:**
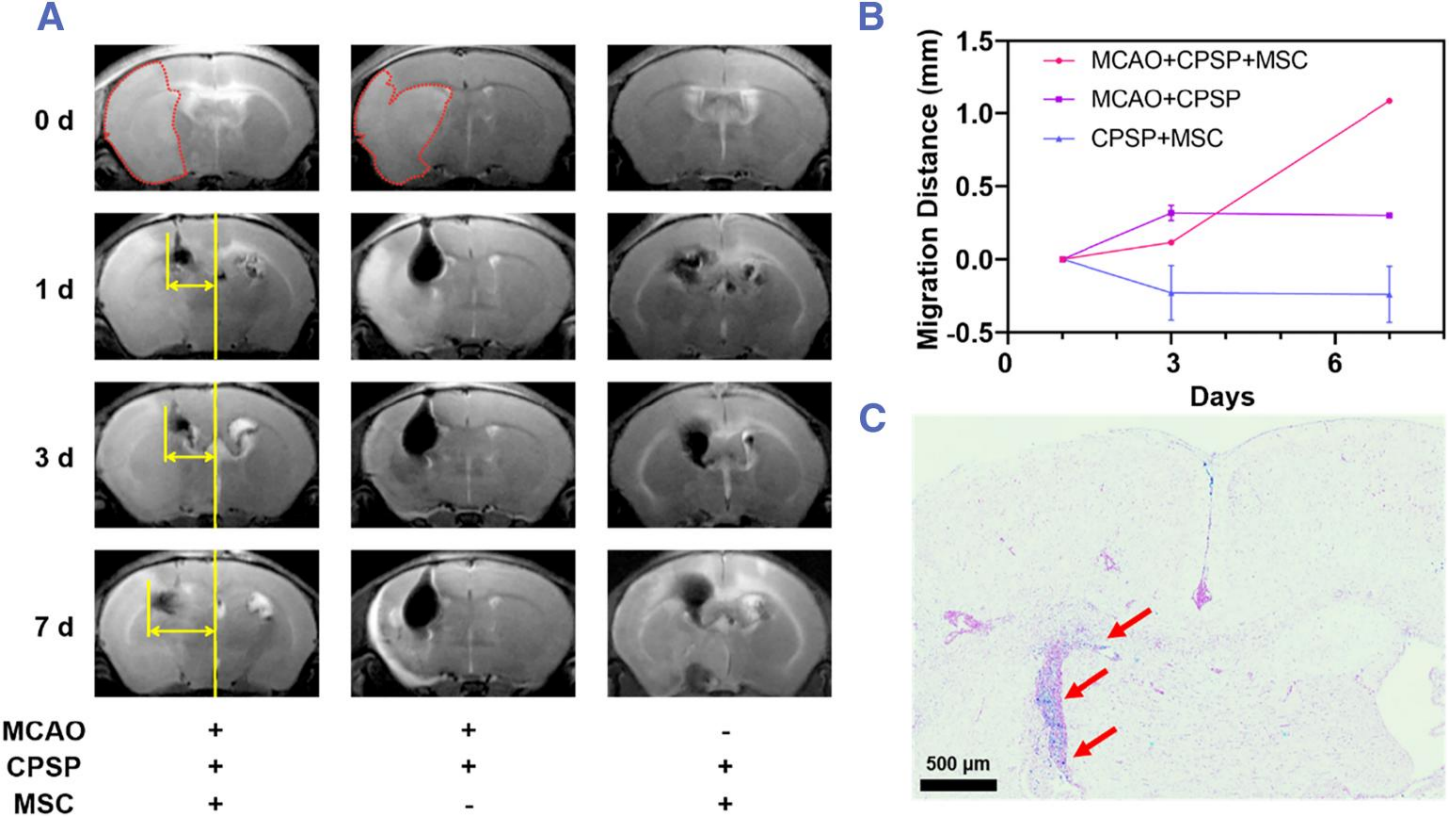
In vivo monitoring of CPSPs and CPSPs‐labeled MSCs via MR imaging. (A) MR images of brains of MCAO mice or healthy mice before and 1, 3, and 7 days after transplantation of CPSP‐labeled MSCs or injection of CPSPs. The areas circled by red dotted lines indicate the ischemic region. The yellow lines with arrows indicate the distance from the lateral edge of the transplanted MSCs to the brain midline. (B) Migration distances of CPSPs or CPSP‐labeled MSCs to the ischemic region counted from (A). (C) Prussian blue staining images of the brain section of MCAO mouse 3 days after transplanted with CPSP‐labeled MSCs. The red arrows indicate the blue‐stained CPSPs. CPSP, cobalt protoporphyrin‐induced nano‐self‐assembly; MCAO, middle cerebral artery occlusion; MR, magnetic resonance; MSC, mesenchymal stem cell.

Although MSCs therapy holds great promise for the treatment of ischemic stroke, spatio‐temporal monitoring of the transplanted MSCs in vivo to thoroughly study their therapeutic mechanism and evaluate their biosafety still remains challenge.^33^, ^34^ MR imaging, as a radiation‐free and a non‐invasive imaging technique, has garnered great attention of the research community in recent years. The high soft tissue contrast and spatiotemporal resolution of the MR imaging make it one of the promising techniques to trace MSCs in brains while simultaneously deciphering the pathological changes within the ischemic stroke. A middle cerebral artery occlusion (MCAO) model was applied to discern the feasibility of CPSPs to monitor MSCs migration in vivo by MR imaging according to the protocol shown in Figure S12. As shown in Figure 5A, the ischemic area with a bright signal at the left hemisphere was clearly visible in the T_2_‐weighted MR imaging at day 0 after the establishment of MCAO, demonstrating the successful construction of the model. The CPSPs, MSCs, and CPSP‐labeled MSCs were then injected to the ischemic perifocal region of MCAO mice or the same location in the brain of normal mice through a stereotaxic method. A clear T_2_‐weighted MR signal was observed from the labeled MSCs and CPSPs in the brains of MACO as well as normal mice after injection (Figure 5A), while no obvious signals could be monitored in the brain of mice transplanted with MSCs alone (Figure S13). More importantly, the labeled MSCs transplanted into the brain of MCAO mouse were observed to migrate to the ischemic region at day 7 after transplantation, while the labeled MSCs transplanted to the brain of normal mouse showed no significant trend of migration (Figure 5A), which could be ascribed to the specific upregulation of stromal cell‐derived factor‐1α protein in the stroke lesion as previously reported.^35^ Meanwhile, the injected CPSPs diffused randomly with the PBS and formed a droplet‐like low signal zone in the ischemic perifocal region. Quantitatively, the migration distances of labeled MSCs to the ischemic lesion areas were measured and found to be 1.08 mm at day 7 after transplantation (Figure 5B). The labeled MSCs, which were stained with Dil before transplantation, could be directly observed in the ischemic perifocal region from the fluorescence images (Figure S14). Meanwhile, the CPSPs could be found at the same location from the Prussian blue staining images, which validated that the T_2_‐weighted MR signal from CPSPs accurately represented the location of the labeled MSCs (Figure 5C).

### 3.6 In vivo therapeutic efficacy of CPSP‐Labeled MSCs on ischemic stroke

**FIGURE 6:**
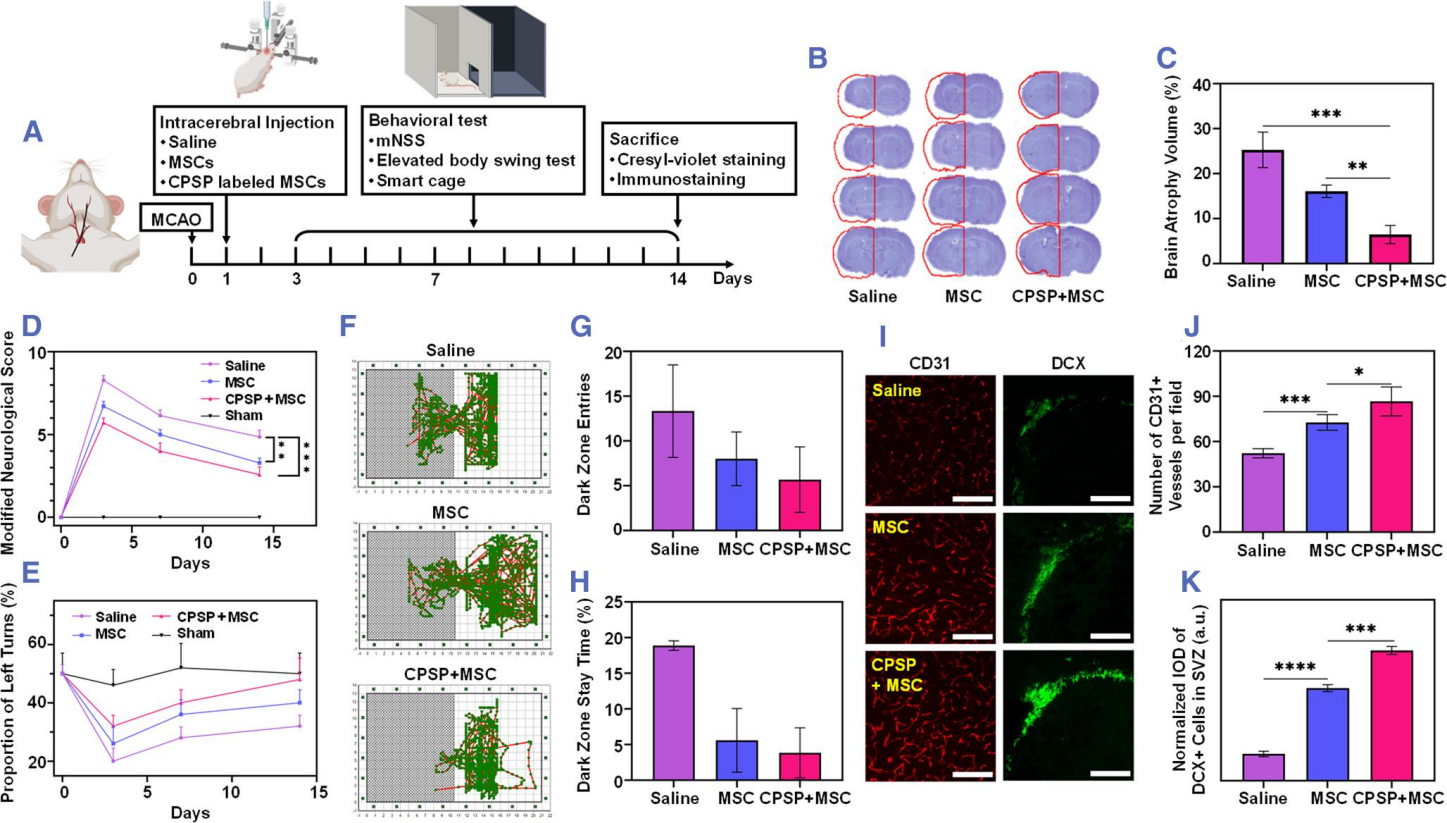
In vivo evaluations of CPSP‐labeled MSCs on ischemic stroke. (A) Experimental protocol for MSC therapy of an MCAO mouse model. (B) Representative set of cresyl‐violet stained coronal brain sections from mice treated with saline, MSCs, and CPSP‐labeled MSCs 14 days after MCAO and (C) the corresponding brain atrophy volume. Brain function recovery was evaluated by (D) neurological score and (E) elevated body swing test. (F) Representative images showed travel patterns of MCAO mice treated with saline, MSCs, and CPSP‐labeled MSCs. (G, H) The frequency that mice entered into the dark zone (G) and the time that mice stayed in the dark zone (H). (I) Representative photographs of CD31 and DCX immunostaining in saline, MSC, and CPSP + MSC groups. (J) Quantification results of CD31^+^ vessels numbers in the ischemic perifocal region. (K) Normalized fluorescence intensity of DCX^+^ cells in the SVZ region. CPSP, cobalt protoporphyrin‐induced nano‐self‐assembly; MCAO, middle cerebral artery occlusion; MSC, mesenchymal stem cell; SVZ, subgranular zone.

The feasibility of CPSP‐labeled MSCs in the ischemic stroke treatment was evaluated by using the MCAO model in accordance with the experimental protocol shown in Figure 6A. The therapeutic effect of CPSP‐labeled MSCs in relieving cerebral atrophy as well as improving neurobehavioral recovery was assessed by Cresyl‐violet staining and behavioral tests, respectively. As shown in Figure 6B,C, the brain atrophy volume was reduced to 16% from 25% after MSC transplantation as compared to the control group, which could further reduce to 6% for the treatment of the CPSP labeling of MSCs. In addition, the neurobehavioral outcome of mice, as an important index for rehabilitation of ischemic stroke, was further evaluated. Similarly as the previous report,^36^ MSC transplantation group shows better behavioral performance of mice in terms of the modified neurologic severity score (Figure 6D), elevated body swing test (Figure 6E), and the step‐through passive avoidance test (Figure 6F–H). More importantly, compared to the MSC group, the behavioral performance of mice transplanted with CPSP‐labeled MSCs was further improved in the above‐mentioned motor and cognitive tests, as confirmed by significantly lower modified neurologic severity scores (mNSS), higher proportions of left turns, less dark zone entries, and less time staying in the dark zone (Figure 6D–H). The above results indicate the significant improvement of the CPSP labeling on the therapeutic effect of the MSC transplantation.

Furthermore, it is reported that transplanted MSCs could promote the neuroprotective effect after stroke by promoting angiogenesis and neurogenesis via paracrine secretion of an array of bioactive factors and neurotrophins.^36^, ^37^ To gain a further insight into the MSCs‐mediated neuronal protection, herein, the endothelial cells of microvessels and neuroblasts origin were stained by CD31 and DCX, respectively, to explore the reparative effects of CPSP‐labeled MSCs on neuroprotective functions. As shown in Figure 6I,J, the numbers of CD31^+^ microvessels were found to have obvious increase in the ischemic perifocal region after transplantation of MSC, which could be further increased by the transplantation of CPSP‐labeled MSCs. Subsequently, the number of DCX^+^ neuroblasts in the subgranular zone (SVZ) of the infarcted hemisphere was evaluated by quantifying the fluorescence area (Figure 6K). The quantitative assessment indicated that the transplantation of CPSP‐labeled MSCs significantly promoted neurogenesis as compared to the MSC group. Collectively, the CPSP‐labeling group greatly enhanced the angiogenesis and neurogenesis effect of the transplanted MSCs in an infarcted stroke model in mice, thus remarkably improving neurological recovery.

## 4 CONCLUSION

In summary, a novel dual‐functional nanoprobe CPSP, which is combined SPIONs with CoPP by a simple solvent evaporation‐induced self‐assembly strategy, was developed for simultaneously tracing and protecting stem cells in vivo. The obtained CPSP shows tunable proportion of active ingredients and good biosafety since there are no additional carrier materials needed during the synthesis. By aggregating small‐sized SPIONs into self‐assembled nanoparticles, the MR imaging performance of CPSP is significantly improved, enabling long‐term in vivo MRI tracking of labeled MSCs. Moreover, the slow release of CoPP wrapped on the surface of CPSP could appropriately upregulate HO‐1 expression of MSCs, which effectively promoted the survival of MSCs in an ischemic stroke environment in mice. Furthermore, administration of CPSP‐labeled MSCs into the brains of ischemic mice was found to significantly decrease the atrophy volume and increase the angiogenesis and neurogenesis, thereby promoting the neurobehavioral recovery. Collectively, this novel nanoprobe endowing with MR imaging and cytoprotective functions presents a novel labeling nanoplatform for MSC therapy. In particular, this easily controlled self‐assembly synthesis method provides a promising strategy to integrate various functional components into one nanoparticle, presenting good potentials for the further clinical translation.

## 5 EXPERIMENTAL SECTION

### 5.1 Chemicals

Iron chloride hexahydrate (FeCl_3_·6H_2_O), oleic acid (OA, technical grade 90%), sodium oleate, 1‐tetradecene (TDE, technical grade 92%), and 1‐octadecene (ODE, technical grade 90%) were purchased form Aladdin. Cobalt protoporphyrin IX (CoPP) was purchased from J&K Scientific. PBS (pH, 7.4) were obtained from Shanghai Runcheng Biomedical Co., Ltd. Fetal bovine serum (FBS), Dulbecco's modified Eagle's medium (DMEM), penicillin, and streptomycin were purchased from Gibco.

### 5.2 Synthesis of SPIONs

Oleic‐acid‐modified SPIONs with the diameter of 4–6 nm were synthesized via pyrolysis.^24^ Firstly, ferric oleate was prepared as a precursor as follows. 1.08 g (4 mmol) FeCl_3_·6H_2_O and 4.87 g (16 mmol) sodium oleate were dissolved in a mixture consisting of 14 ml of hexane, 8 ml of ethanol, and 6 ml of water. After re‐fluxing for 6 h at 70°C with magnetic stirring at 350 rpm, the ferric oleate was obtained, which was washed with ethanol three times. Thereafter, 0.9 g (1 mmol) of iron oleate precursor was dissolved in a mixture of 142 mg (0.5 mmol) OA, 1.75 g (9 mmol) TDE, and 3.25 g (13 mmol) ODE and heated to 300°C for 1 h. Finally, SPIONs were precipitated by adding ethanol and dispersing in toluene as a stock solution.

### 5.3 Synthesis of CPSPs

1.5 mg of CoPP was dissolved in 50 μL dimethyl sulfoxide (DMSO) followed by the addition of 150 μL SPIONs‐dispersed toluene solution (15 mg/ml). The mixture was vortexed at 50°C to homogenously mix CoPP and SPION. The mixture was then added to a glass vial containing 8 ml of water. Next, this mixed solution with stratification was subjected to sonication by a probe at power of 600 W until a homogeneous solution was obtained. The glass vial was left open and placed in a dark ventilated environment overnight to ensure the complete evaporation of toluene. Dialysis was performed overnight to remove unloaded CoPP and DMSO. The formed CPSPs were then collected by centrifugation at 13,000 rpm for 15 min and extensively washed.

### 5.4 Synthesis of h‐SPIONs

The oleic acid‐modified SPIONs were transferred to an aqueous phase following a previous study.^30^ About 5 mg of oleic acid‐modified SPIONs and 15 μL oleic acid were added into 5 ml hexane and sonicated for 1 min to afford an even dispersion. Subsequently, the mixed solution was added to 25 ml water and sonicated by using an ultrasonic probe at power of 300 W for 5 min until the formation of homogeneous emulsion, which was allowed to settle for 1 day. The h‐SPIONs were obtained by collecting the colored aqueous fraction in the bottom of the separated emulsion.

### 5.5 Characterizations

The hydrodynamic sizes were evaluated by DLS (Malvern, Nano ZS90). The morphology of materials and cells as well as element mapping images of material samples were acquired by TEM (JEM‐2100F). The surface morphology of material was obtained by SEM (Magellan400). The relaxation time of the material was evaluated by an NMI20‐015V‐I nuclear magnetic resonance analyzer. The elemental quantitative analyses were performed with inductively coupled plasma optical spectrometry (ICP‐OES). The UV‐vis spectra of materials were acquired by a spectrophotometer (Shimadzu UV‐3600). Fourier transform infrared spectroscopy analyses were carried out with a spectrometer (BRUKER, ALPHA II). Energy‐dispersive X‐ray spectra of samples were obtained by an X‐ray photoelectron spectroscope (ESCALAB 250).

### 5.6 Cell culture and labeling

All animal protocols were approved by the Institutional Animal Care and Use Committee, Shanghai Jiao Tong University. MSCs were isolated from adult male Sprague‐Dawley rats (Sippr‐BK Co.) weighing 200 g according to a previously report.^38^ Bone marrow MSCs were cultured in a 10‐cm dish (Corning Incorporated) containing 6 ml culture medium at 37°C and 5% CO_2_ in an incubator (Thermo Scientific). The culture medium was composed of high‐glucose Dulbecco's modified Eagle's medium (DMEM; Gibco), 10% v/v fetal bovine serum (FBS, Gibco), 100 unit/ml penicillin, and 100 mg/ml streptomycin. MSCs were isolated by their adherence to culture plastic. The nonadherent cells were removed and adherent cells were further cultured. The culture medium was changed every 3 days. MSCs between passages 3 and 5 were used in the study.

To label MSCs with CPSPs, different concentrations of CPSPs were added to the culture medium. The corresponding magnets (grade N35 NdFeB with diameter of 100 mm and height of 10 mm for 10‐cm dish; 96‐well magnetic plate [NANOEAST] for 96‐well plate) were placed under the culture medium for 20 min to promote the internalization of the CPSPs, and the MSCs were then incubated for another 6 h of co‐incubation. The MSCs were washed by PBS for several times to ensure the removal of extracellular CPSPs. Fresh culture medium was then added and the labeled MSCs were cultured for further experiment. The content of the CPSPs internalized by the MSCs was characterized by ICP‐OES and Prussian blue staining as previously reported.^39^

### 5.7 In vitro antioxidative stress

To evaluate the hypoxia relief capacity of CPSPs, an OGD study was performed. MSCs labeled with different concentrations of CPSPs or untreated MSCs were simultaneously cultured with DMEM free of glucose and FBS.^40^ The plates were placed into an anaerobic chamber using a well‐characterized, finely controlled modular chamber system (Billups‐Rothenberg, Inc.), and flushed with a mixture gas of 95% N_2_/5% CO_2_ at 10 l/min for 30 min at room temperature. Then, the chamber was sealed and placed in a conventional cell incubator at 37°C for 12 h. Next, the medium was replaced with FBS‐containing/high‐glucose DMEM and the MSCs were returned to the normal incubator environment (37°C/20% O_2_/5% CO_2_) for 12 h of reoxygenation procedure. Untreated MSCs were kept for the same time in the 10% FBS‐containing DMEM solution under a normoxic atmosphere. The cell viability was tested by a CCK‐8 assay, and the magnitude of HO‐1 induction in MSCs was evaluated by Western blotting analysis.

### 5.8 Western blotting

The HO‐1 expression of MSCs was measured through western blotting. The protein of the MSCs was obtained by lysis, followed by quantifying the concentration of protein via BCA kit. Subsequently, the same amount of protein from each sample was separated by 12% w/v SDS‐PAGE gel electrophoresis and electrotransferred to the PVDF membrane. The nonspecific protein was blocked by 5% skim milk at room temperature for 1 h, and the membranes were then probed with GAPDH and HO‐1 antibodies at 4°C overnight. After washed with Tris‐buffered saline and Tween‐20 for three times, the membranes were incubated with horseradish peroxidase‐conjugated anti‐mouse for 1 h at room temperature, and the protein expression was detected by an enhanced electrochemiluminescence substrate. The result was semi‐quantified by ImageJ.

### 5.9 Establishment of the middle cerebral artery occlusion mouse model

Adult male ICR mice weighting 25–30 g were used to establish the MCAO mouse model. Mice were anaesthetized with 1.5%–2% isoflurane and 70/30% nitrous oxide/oxygen, while the body temperature was maintained at around 37°C by applying a heating pad. Briefly, the common carotid artery (CCA), internal carotid artery (ICA), and external carotid artery (ECA) were carefully separated. Subsequently, a 6‐0 suture coated with silicon was gently inserted into the middle cerebral artery pass through the intersection of the ECA and the ICA. The successful occlusion was confirmed by monitoring the decrease in surface cerebral flow to 10% of baseline. Reperfusion was conducted by withdrawing the suture 90 min after the occlusion.

### 5.10 MR imaging in vitro and in vivo

To evaluate the in vitro MR capability of the SPIONs in different formulations, SPIONs and CPSPs were dissolved in water to obtain five solutions with a certain concentration gradient of iron in the range of 0–0.32 mmol/L. The liquid samples were placed in chromatography vials, and their T_2_‐weighted images were obtained by a 0.5 T MRI scanner at room temperature. Relaxivity (*r*_2_) values were calculated via fitting the curve of 1/T_2_ relaxation time (s^−1^) versus the concentration of Fe (mM). The concentration of Fe was determined by ICP‐OES.

After 24 h of MACO, mice were divided into three groups with five mice in each group. Unlabeled MSCs, CPSP‐labeled MSCs (1 × 10^6^ MSCs per mouse), and CPSPs were dispersed in 50 μL PBS and injected into the striatum of the infarcted hemisphere of mice with the following coordinates: AP—0.5 mm; L—1.5 mm; V—2.0 mm. In vivo MR imaging was conducted before and at day 1, day 3, and day 7 after transplantation by a 7 T MRI scanner. A T_2_‐weighted fast spin echo sequence was used with the following parameters: TR = 2500 ms, TE = 33 ms, and slice thickness = 1.2 mm.

### 5.11 Neurobehavioral tests

mNSS, including reflex, motor, and balance tests, were performed before MCAO and at days 3, 7, and 14 after MCAO. The mNSS of the mice was graded from 0 to 14, where 0 represents normal and a higher score indicates more severe injury.

The elevated body swing test was performed to test asymmetrical motor behavior. Mice were held by the base of their tails and elevated to 10 cm above the surface where they were resting. The initial swing direction, defined as the turning of the upper body by >10° from the vertical axis, was recorded in 20 trials of each rat. The total number of swings to each (left or right) side was counted for each rat.

Step‐through passive avoidance test was conducted to evaluate the learning and memory ability of the mice by using a smart cage purchased from AfaSci. The smart cage was consisted with a dark chamber and a bright chamber, which were connected through a door. During training, the mice were subjected an electric shock on their foot while they were entering the dark chamber. Twenty four hour after training, the number of entries into the dark chamber and the time spent in the dark chamber of the mice were recorded for 10 min without electric shock.

### 5.12 Prussian blue staining, cresyl violet staining, and immunofluorescence analysis

The brain of the MCAO mouse was fixed via transcardial perfusion with 4% PFA 3 days after transplanted with CPSP‐labeled MSCs. After perfusion, the brain was removed and embedded in paraffin, followed by Prussian blue staining as previously described.^39^

For cresyl violet staining and immunofluorescence, the brains of mice in each group were removed and frozen after perfusion at day 14 after transplantation. The 10‐μm‐thick sections of the brains from anterior commissure to hippocampus were collected and stained by 0.1% cresyl violet. The atrophy volume was calculated by subtracting the volume of the ipsilateral hemisphere from the contralateral hemisphere volume.

The apoptosis of transplanted MSCs was estimated through terminal deoxynucleotidyl transferase dUTPnick‐end labeling (TUNEL) staining as previously reported.^40^ Angiogenesis and neurogenesis were evaluated by immunofluorescence. Briefly, the brain sections were blocked using bovine serum albumin and incubated with goat anti‐CD31 or goat anti‐doublecortin (DCX) at 4°C overnight. After washing with PBS, the sections were incubated with Alexa Fluor 488‐conjugated donkey anti‐goat or Alexa Fluor 647‐conjugated donkey anti‐goat. The stained sections were observed by a fluorescence microscope.

## AUTHOR CONTRIBUTIONS

Yimeng Shu: Methodology; Validation; Formal analysis; Investigation; Visualization; Writing – Original Draft. Hui Shen: Data Curation; Resources; Validation. Minghua Yao: Investigation; Resources; Funding acquisition. Jie Shen: Investigation; Data Curation. Guo‐yuan Yang: Conceptualization; Resources. Hangrong Chen: Writing – Review and Editing; Funding acquisition. Yaohui Tang: Supervision; Writing – Review & Editing; Funding acquisition. Ming Ma: Supervision; Conceptualization; Writing – Review and Editing; Funding acquisition.

## CONFLICT OF INTEREST STATEMENT

The authors declare that they have no known competing financial interests or personal relationships that could have appeared to influence the work reported in this paper.

## ETHICS STATEMENT

This study was approved by the Committee on the Ethics of Animal Experiments of Shanghai Jiao Tong University (Approval No. 2020033). All animal protocols were authorized by the Institutional Animal Care and Use Committee, Shanghai Jiao Tong University.

## Supporting information

Supporting Information S1
