# Supplementary material for: Metal protoporphyrin‐induced self‐assembly nanoprobe enabling precise tracking and antioxidant protection of stem cells for ischemic stroke therapy
Source: Smart Med. 2023 Feb 14;2(1):e20220037. doi: 10.1002/SMMD.20220037 (PMC11236039; doi:10.1002/SMMD.20220037)
Supplement: Supplementary file 1 — Supporting Information S1 [file SMMD-2-e20220037-s001.docx]

Supporting Information

**Metal Protoporphyrin-Induced Self-Assembly Nanoprobe Enabling Precise Tracking and Antioxidant Protection of Stem Cells for Ischemic Stroke Therapy**

# *Yimeng Shu, Hui Shen, Minghua Yao, Jie Shen, Guo-Yuan Yang, Hangrong Chen, Yaohui Tang*, and Ming Ma**

# Supplementary Figures
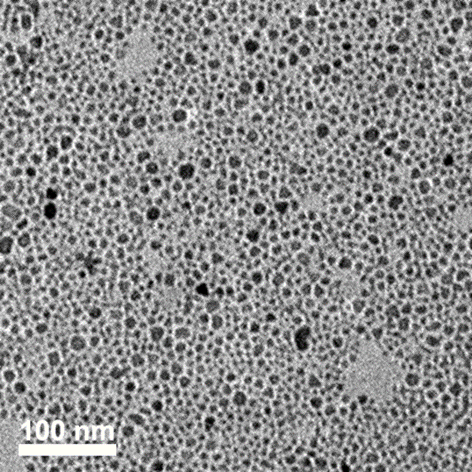


**Figure S1.** TEM image of synthesized SPIONs.

**
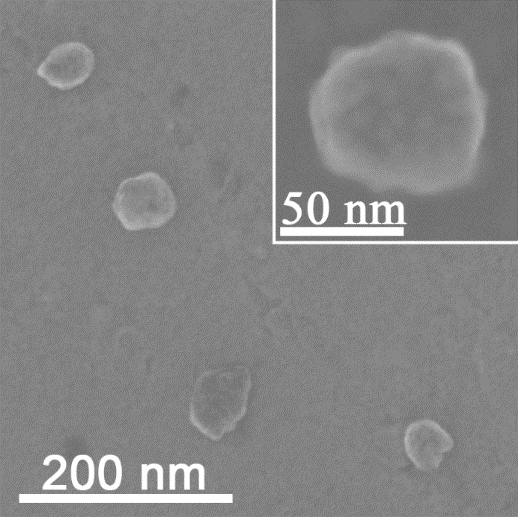
**

**Figure S2.** SEM image of CPSPs at different magnifications.

**
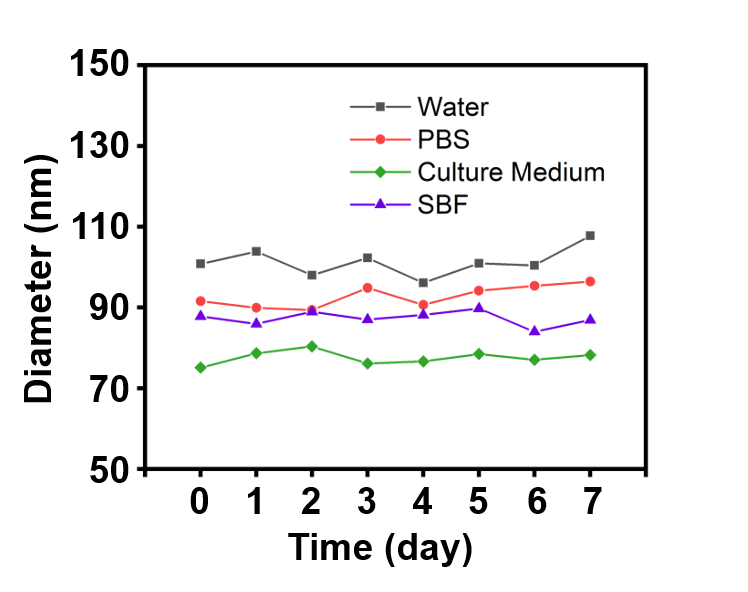
Figure S3.** Hydrodynamic diameter of CPSP in water, PBS, culture medium, and simulated body fluid (SBF) for up to 7 days.


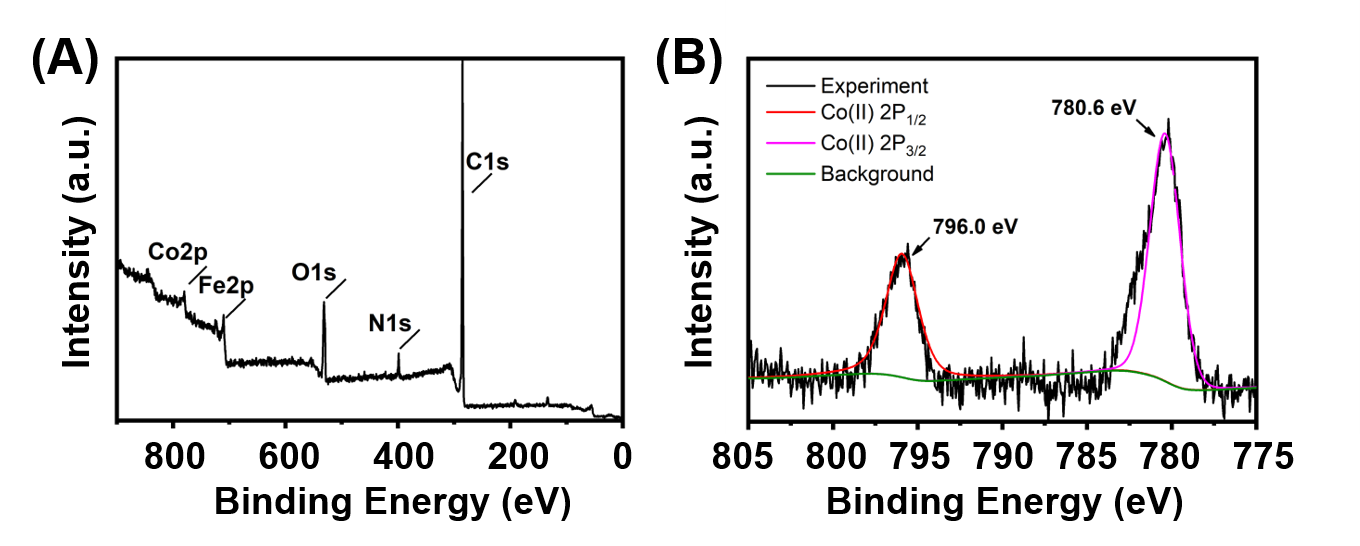


**Figure S4.** (A) XPS spectrum of CPSP, and (B) the corresponding high-resolution XPS spectrum in the Co2p region.


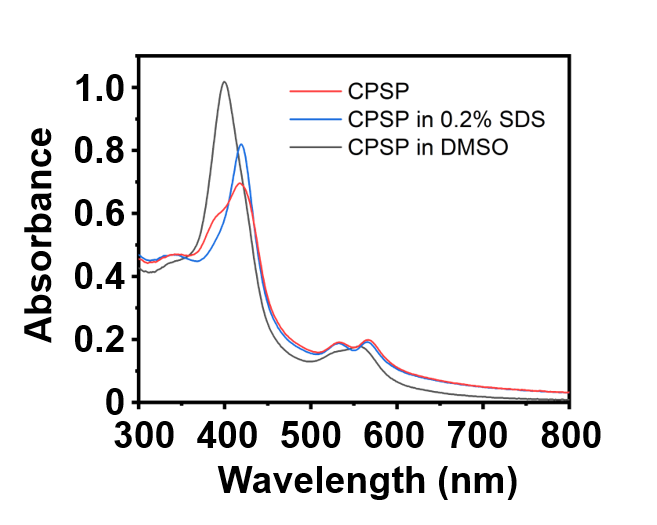


**Figure S5.** UV-vis spectra of CPSP in water, 0.2% SDS (w/v), and DMSO.


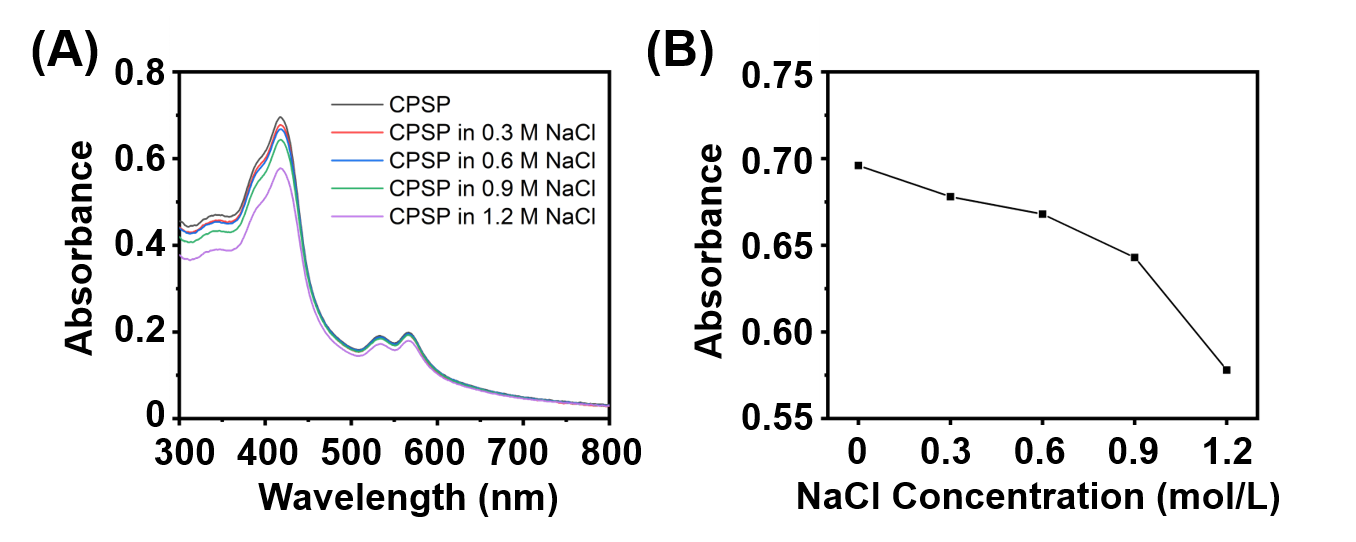


**Figure S6.** (A) UV-vis spectra of CPSP in water and NaCl solutions at different concentrations, and (B) the corresponding absorbance value at 417 nm.


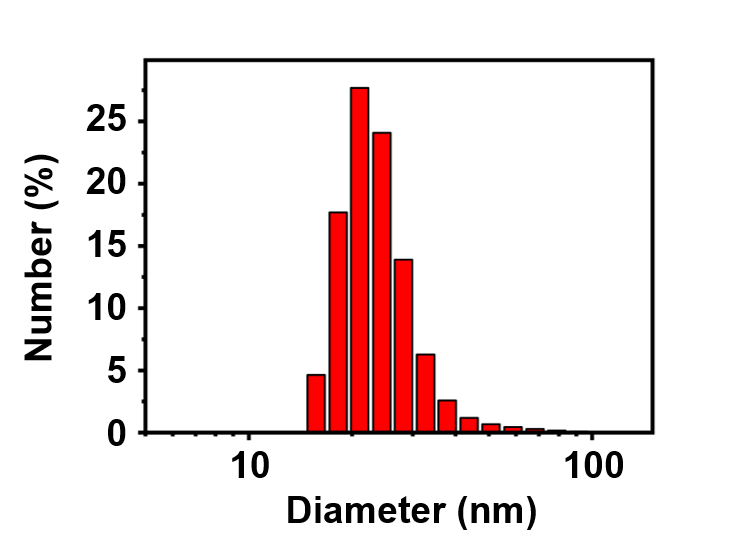


**Figure S7.** Hydrodynamic diameter of h-SPIONs.


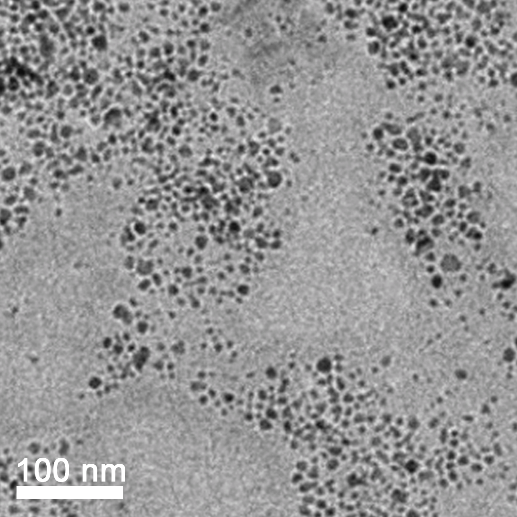


**Figure S8.** TEM image of synthesized h-SPIONs.


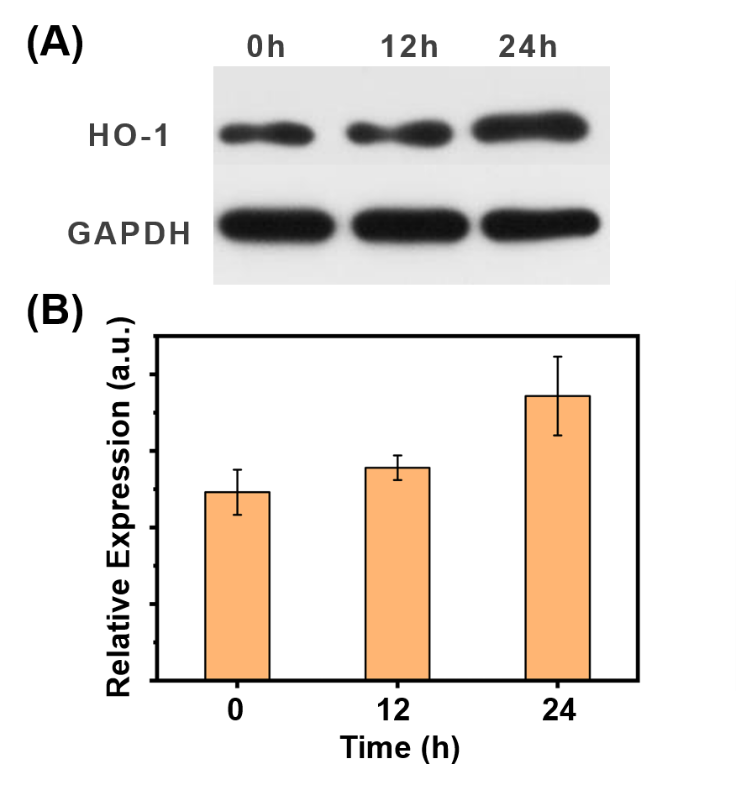


**Figure S9.** (A) HO-1 western blot expression levels and (B) the corresponding semiquantative data of untreated MSCs and MSCs treated with 5 μg CPSP for 12 h and 24 h.


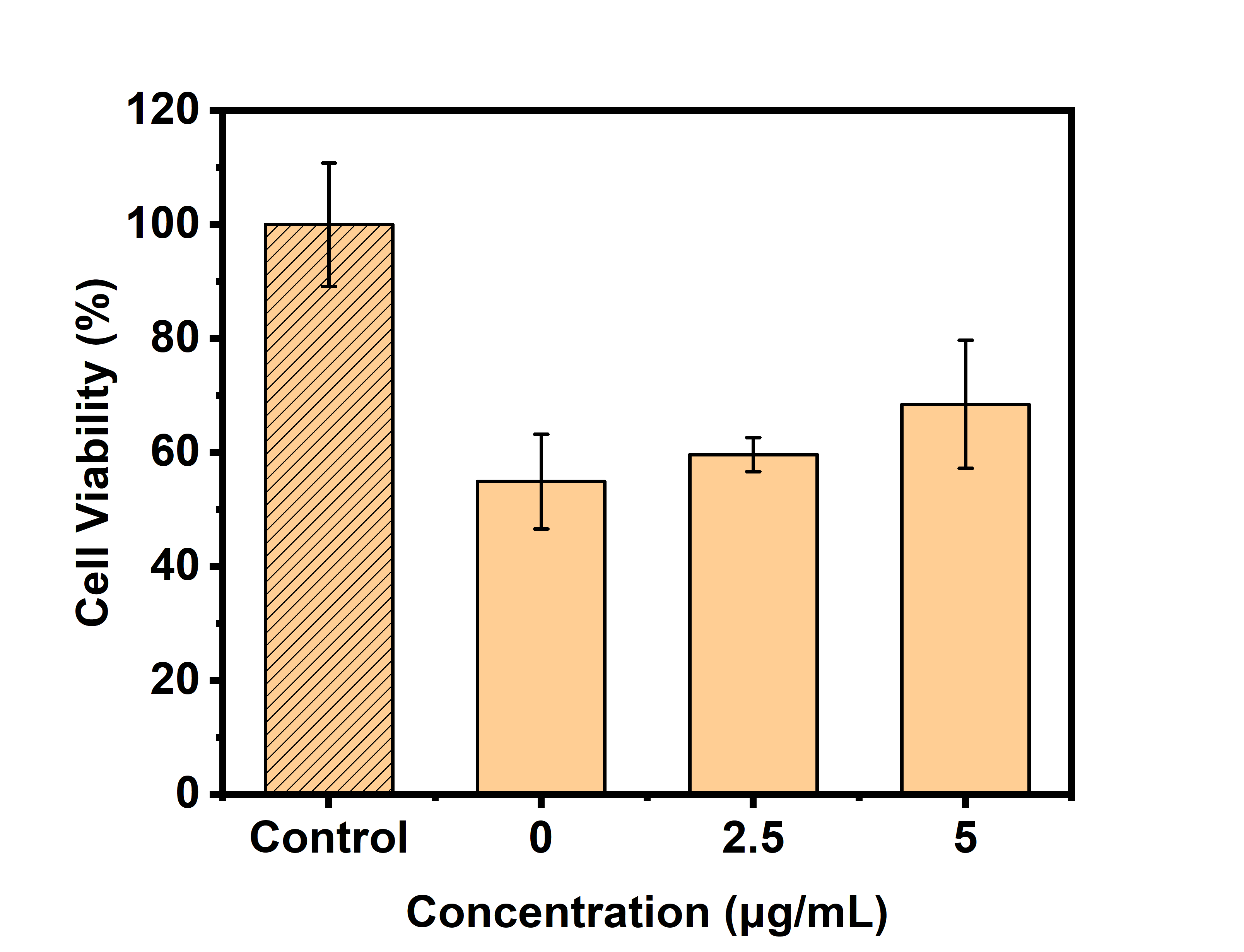


**Figure S10.** Cell viability of unlabeled MSCs and MSCs labeled by CPSPs at different concentrations in H_2_O_2_-induced oxidative stress mode.


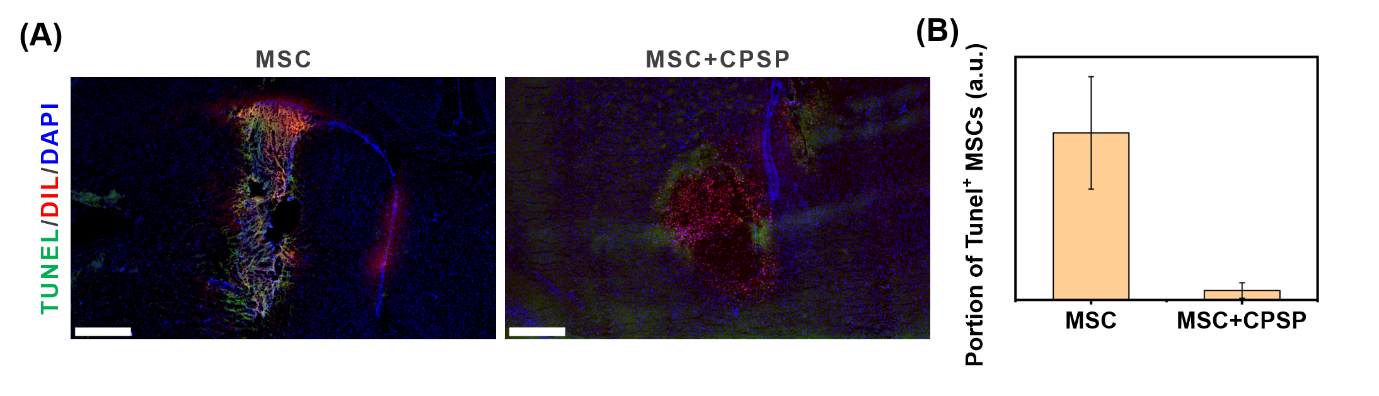


**Figure S11.** (A) Representative TUNEL staining images of the brains sections of ischemia mice transplanted with MSCs and CPSP labeled MSCs and (B) the corresponding portions of TUNEL+ MSCs. Scale bar, 500 μm.


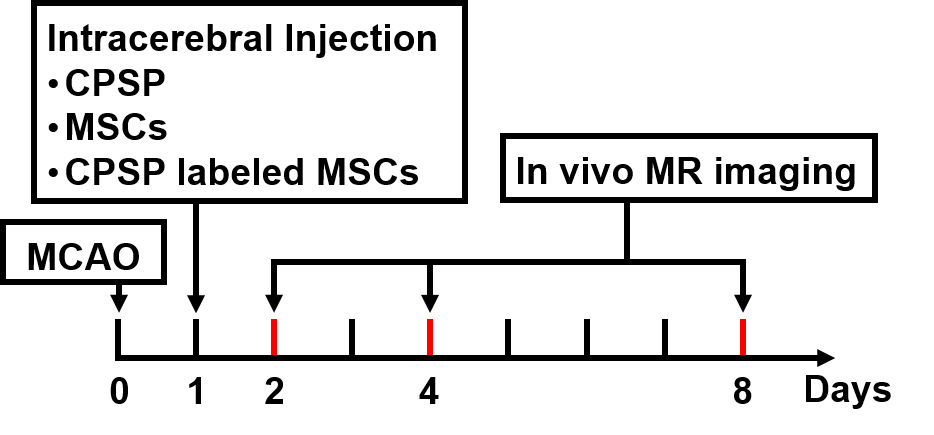


**Figure S12.** Experimental protocol for tracking MSC with MR imaging in a MCAO model.


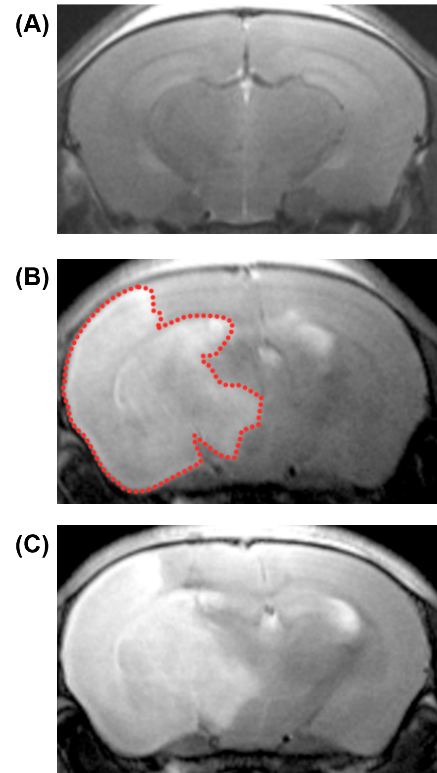


**Figure S13.** MR images of the mouse brain (A) before MCAO, (B) 1 day after MCAO, and (C) 1 day after MSCs transplantation. The areas circled by red dotted lines indicate the ischemic region.


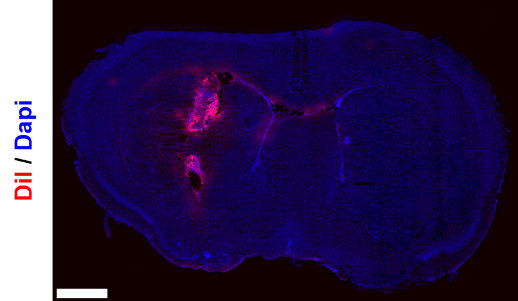


**Figure S14.** Fluorescence image of brain section 3 days after transplantation of MSCs labeled with both CPSPs and Dil. Scale bar, 1000 μm.
